# Braincase anatomy of the Paleocene crocodyliform *Rhabdognathus* revealed through high resolution computed tomography

**DOI:** 10.7717/peerj.11253

**Published:** 2021-05-04

**Authors:** Arthur Erb, Alan H. Turner

**Affiliations:** Department of Anatomical Sciences, Stony Brook University, Stony Brook, NY, United States of America

**Keywords:** Crocodylomorpha, Dyrosauridae, Archosauria, Three-dimensional, Endocast, Neuroanatomy, Computed tomography

## Abstract

Dyrosaurids were highly specialized, largely marine, relatives of living crocodylians, and one of the few archosaur lineages to survive the K-Pg extinction. Dyrosaurids lived during the Cretaceous to the Eocene and represent a unique combination of morphology and ecology not seen in living crocodylians. Little is known about their endocranial anatomy, leaving many questions about their neurosensory adaptations unaddressed. Recently, µCT (micro-computed tomography) scans were made of a well-preserved skull of *Rhabdognathus*, a Paleocene dyrosaurid from Mali. This marks the first time the braincase and neurosensory features of a dyrosaurid have been examined using CT. We focus our attention to three specific internal structures: the cranial endocast; the inner ear; and the paratympanic sinuses. The cranial endocast of *Rhabdognathus* revealed novel features including a unique conformation of its paratympanic system, a prominent dorsal venous system that communicates with the external skull table, extremely enlarged tympanic vestibules that meet at the midline of the endocranium, a prominent spherical cerebrum, and elongate olfactory tracts accounting for half the total endocast length. The bizarre laterally facing lateral Eustachian foramen of dyrosaurids is now understood to be a complex fossa including both a ventrally directed lateral Eustachian foramen and a laterally directed foramen for the basioccipital diverticulum. A novel median pterygopharyngeal canal was discovered connecting the pharynx to the adductor chamber. These revelations require a reinterpretation of the associated external foramina visible on the posterior of the skull in dyrosaurids and potentially their close relatives the pholidosaurids. The olfactory tract terminates in an enlarged olfactory region possessing complex bony projections—a unique morphology perhaps serving to increase surface area for olfaction. The inner ear of *Rhabdognathus* exhibits characteristics seen in both *Pelagosaurus* and *Gavialis*. The vestibule is spherical, as in *Gavialis*, but is significantly expanded. The semicircular canals are enlarged but pyramidal in shape as in the thalattosuchian *Pelagosaurus*. The proportion of the cochlear length to total endosseous labyrinth height is roughly 0.5 in *Rhabdognathus* implying that the hearing capabilities resemble that of thalattosuchians. A suite of expanded sense organs (e.g., bony olfactory lamina; hypertrophied vestibule of the inner ear), and the clear expansion of the cerebrum to a more symmetrical and spherical shape suggest that dyrosaurids possess neuroanatomical modifications facilitating an agile predatory near-shore ecology.

## Introduction

Modern crocodylians are relatively conservative in their morphology and ecological preferences, whereas multiple extinct crocodylomorph lineages exhibit much greater diversity in form and ecology (Bronzati, Montefeltro & Langer, 2015; Godoy et al., 2019; Melstrom & Irmis, 2019; Wilberg, Turner & Brochu, 2019). One such lineage is the Dyrosauridae, a clade of extinct crocodylomorphs that survived from the Late Cretaceous through the Late Eocene. Marked by their longirostrine skull morphology, dyrosaurids exhibit a morphology that appears adapted to a more aquatic lifestyle than modern crocodylians, and are found in both near-shore marine and freshwater deposits (Pomel, 1894; Troxell, 1925; Swinton, 1930; Arambourg, 1952; Bergounioux, 1956; Halstead, 1975; Buffetaut & Wouters, 1979; Buffetaut, 1980; Jouve et al., 2005; Barbosa, Kellner & Viana, 2008; Hastings et al., 2010; Hastings, Bloch & Jaramillo, 2011; Hastings, Bloch & Jaramillo, 2015). The earliest dyrosaurids are found in Maastrichtian-aged marginal marine sediments of Africa, India, and Central America (Hill et al., 2008; Khosla et al., 2009; Salih et al., 2016; Shiller, Porras-Muzquiz & Lehman, 2016) and potentially Cenomanian deposits in Europe (Buffetaut & Lauverjat, 1978). The clade rapidly diversified and is found in Paleogene deposits formed from the Tethys Sea in North America (Troxell, 1925), which suggests an early transatlantic dispersal.

**Figure 1:**
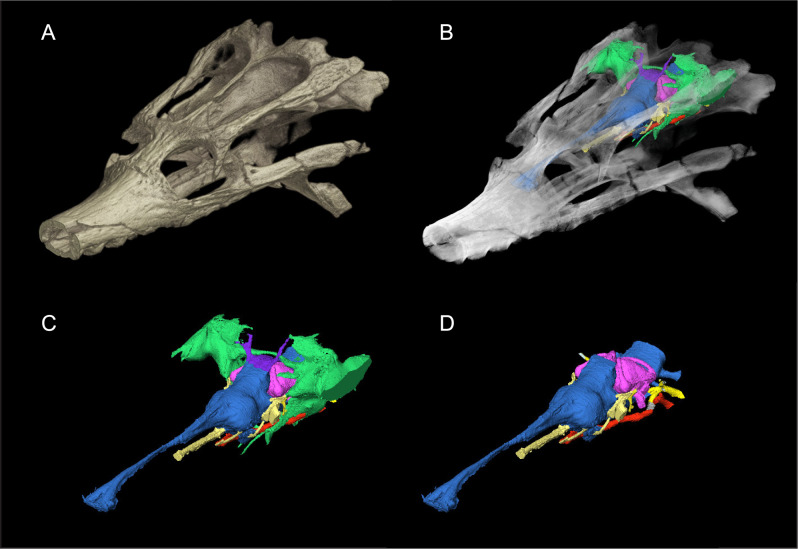
Skull of *Rhabdognathus aslerensis* (AMNH FARB 33354). (A) Three-dimensional (3D) digital model. (B) Semi-transparent 3D model with underlying endocranial anatomy. (C) Isolated endocranial anatomy. (D) Isolated endocranial anatomy with paratympanic system removed.

The dyrosaurid *Rhabdognathus* first appears in the Late Cretaceous of Africa and survived into the Paleogene, making it one of the few crocodylomorph genera to survive the K-Pg extinction. The original type species of the genus, *R. rarus* (Swinton, 1930), was based on fragmentary mandibular remains and was later found to be a nomen dubium (Jouve, 2007). Two species are currently recognized, *R. aslerensis* (Jouve, 2007) and *R. keiniensis* (Jouve, 2007), both based on cranial remains from Mali. The type specimen of *R. aslerensis* (AMNH FARB 33354, American Museum of Natural History, Fossil Amphibians, Reptiles, and Birds) was originally referred to as cf. *Rhabdognathus* by Brochu et al. (2002) in their description of it. This specimen is a nearly complete skull missing only the most anterior tip of the snout. Its exceptional three-dimensional preservation makes it ideal for investigating dyrosaurid endocranial anatomy (Fig. 1).

Currently, no dyrosaurid endocasts have been described leaving a gap in the contemporary understanding of variation and potential adaptations in crocodile-line archosaur neuroanatomy. The majority of CT-based endocast descriptions of longirostrine archosaurs are of metriorhynchids (Fernández et al., 2011; Herrera & Vennari, 2015; Brusatte et al., 2016; Pierce, Williams & Benson, 2017; Herrera, Leardi & Fernández, 2018), phytosaurs (Holloway, Claeson & O’keefe, 2013; Lautenschlager & Butler, 2016), or the extant crocodylian *Gavialis* (Bona, Carabajal & Gasparini, 2017; Pierce, Williams & Benson, 2017). These studies complement the older paleoneurology literature based on natural endocasts (e.g., Koken, 1893; Edinger, 1938; Edinger, 1975; Wenz, 1968; Hopson, 1977; Hopson, 1979). Despite the similarities in skull morphology, metriorhynchids differ from dyrosaurids in being pelagic, fully marine forms. Here we provide a detailed anatomical description of the endocranial anatomy of *Rhabdognathus* based on high resolution µCT data. These data reveal novel morphologies in the endosseus labyrinth, paratympanic sinuses, and olfactory region that may relate to dyrosaurid adaptations to transitional marine habitats.

## Material and Methods

The specimen, AMNH FARB 33354, comes from the Pgi rocks of Mali. The geologic setting and external skull morphology of this specimen was previously described by Brochu et al. (2002). Thus the primary focus here is on the endocranial anatomy. AMNH FARB 33354 was µCT scanned on a GE v—tome—x system at the American Museum of Natural History with a voltage of 200 kv, current of 250 µa, and a 0.5 mm copper filter acquiring 1,500 images with an isometric voxel size of 91 µm. Three dimensional digital models were created for the endocranial space, endosseous labyrinth, and pharyngotympanic space using manual image segmentation in Avizo v7 (ThermoFisher) and following best practices outlined in Balanoff et al. (2016). Due to the marked difference in density between the fossil bone and matrix, discerning endocranial elements required infrequent and slight adjustment of grayscale thresholds. The brush tool was used to select the endocranial space at bone boundaries and the space within the boundary captured using the fill command.

Comparisons with the endocasts of previously described crocodile-line archosaurs were made in order to better understand the potential endocranial adaptations present in *Rhabdognathus*. Our comparative sample consisted of 25 crocodile-line archosaurs (Table 1). Morphometric data for these endocasts was obtained via direct measurement of specimen images in ImageJ64 and from Pierce, Williams & Benson (2017) (Table 2).

**Table 1:** Comparative sample of 25 crocodile-line archosaurs used throughout the description together with the source used for their endocranial morphology.

**Species**	**Source**
*Aegisuchus witmeri* (Neosuchia)	Holliday & Gardner (2012)
*Alligator mississippiensis* (Crocodylia)	Witmer & Ridgely (2008)
*Allodaposuchus hulki* (Neosuchia)	Blanco et al. (2015)
*Caiman crocodilus* (Crocodylia)	Jirak & Janacek (2017)
*Crocodylus johnstoni* (Crocodylia)	Witmer et al. (2008)
*Crocodylus niloticus* (Crocodylia)	Jirak & Janacek (2017)
*Cricosaurus araucanensis* (Thalattosuchia)	Herrera, Leardi & Fernández (2018)
*Dakosaurus cf. andiniensis* (Thalattosuchia)	Herrera & Vennari (2015)
*Desmatosuchus spurensis* (Aetosauria)	Hopson (1979)
*Ebrachosuchus neukami* (Phytosauria)	Lautenschlager & Butler (2016)
*Gavialis gangeticus* (Crocodylia)	Pierce, Williams & Benson (2017)
*Goniopholis pugnax* (Goniopholididae)	Edinger (1938)
*Lohuecosuchus megodontos* (Neosuchia)	Serrano-Martínez et al. (2019)
*Machaeroprosopus mccauleyi* (Phytosauria)	Holloway, Claeson & O’keefe (2013)
*Metriorhynchus cf. westermanni* (Thalattosuchia)	Fernández et al. (2011)
*Parasuchus angustifrons* (Phytosauria)	Lautenschlager & Butler (2016)
*Parringtonia gracilis* (Erpetosuchidae)	Nesbitt et al. (2017)
*Pelagosaurus typus* (Thalattosuchia)	Pierce, Williams & Benson (2017)
*Pholidosaurus meyeri* (Pholidosauridae)	Edinger (1938) and Hopson (1979)
*Prestosuchus chiniquensis* (Loricata)	Mastrantonio et al. (2019)
*Rukwasuchus yajabaliijekundu* (Notosuchia)	Sertich & O’Connor (2014)
*Sebecus icaeorhinus* (Notosuchia)	Colbert, Simpson & Williams (1946) and Hopson (1979)
*Simosuchus clarki* (Notosuchia)	Kley et al. (2010)
*Steneosaurus cf. gracilirostris* (Thalattosuchia)	Brusatte et al. (2016)
*Steneosaurus bollensis* (Thalattosuchia)	Herrera, Leardi & Fernández (2018)

**Table 2:** Linear morphometric data. Selected measurements of endocasts and bony labyrinths among pseudosuchian archosaurs. Choice of measurements based on (Pierce, Williams & Benson, 2017). Data for taxa marked by asterisk from Pierce, Williams & Benson (2017). Measurements presented in millimeters or degrees.

**Taxon**	**SW**	**CFA**	**PFA**	**EL**	**OL**	**CW**	**PW**	**PH**	**PL**	**LH**	**LW**
*Aegisuchus witmeri*	?	?	?	?	?	?	?	?	?	?	?
Alligator mississippiensis*	73	135	145	98	48	21	5	8	10	18	14
*Allodaposuchus hulki*	?	168	?	95	47	24	?	?	?	?	?
*Caiman crocodilus*	?	138	162	71	34	21	?	?	?	?	?
*Cricosaurus araucanensis*	100	166	165	141	83	26	12	9	15	19.5	18
Crocodylus johnstoni*	?	145	153	103	46	29	5	8	11	13	14
*Crocodylus niloticus*	?	152	160	113	58	33	?	?	?	?	?
*Dakosaurus cf. andiniensis*	?	?	?	?	?	?	?	?	?	?	?
Desmatosuchus spurensis*	?	132	131	117	30	34	?	11	12	?	?
Ebrachosaurus neukami*	101	147	146	100	53	18	?	12	7	14	18
Gavialis gangeticus*	168	150	154	146	55	32	6	9	11	21	21
Goniopholis pugnax*	?	140	161	117	42	31	15	?	?	?	?
*Lohuecosuchus megodontos*	?	166	174	124	51	22	6	6	7	11	7
*Machaeroprosopus mccauleyi*	150	133	141	138	71	20	?	?	?	?	?
*Metriorynchus cf. westermanni*	?	?	?	?	?	?	?	?	?	?	?
Parasuchus angustifrons*	78	137	136	95	47	19	?	11	8	14	18
*Parringtonia gracilis*	?	134	157	?	?	?	?	?	?	?	?
Pelagosaurus typus*	52	160	160	57	21	15	6	7	10	14	11
Pholidosaurus meyeri*	?	143	150	138	51	28	12	9	20	?	?
*Prestosuchus chiniquensis*	?	115	?	?	?	?	?	?	?	?	?
*Rhabdognathus aslerensis*	89	158	152	171	104	26	5	10	14	25	26
*Rukwasuchus yajabalijekundu*	?	152	158	104	49	30	8	7	4	?	?
Sebecus icaeorhinus*	147	150	160	120	46	30	?	9	8	?	?
Simosuchus clarki*	58	142	165	79	25	25	5	9	10	?	?
*Steneosaurus bollensis*	?	170	165	76	26	20	4	6	4.5	20.5	16
Steneosaurus cf. gracilirostris*	?	175	170	?	?	28	14	12	17	26	26

**Taxon**	**CL**	**ASCA**	**PSCA**	**LSCA**	**CW:SW**	**CW:EL**	**OL:EL**	**PW:PH**	**PW:PL**
*Aegisuchus witmeri*	?	?	?	?	?	?	?	?	?
Alligator mississippiensis*	8	35	12	13	0.29	0.21	0.49	0.63	0.5
*Allodaposuchus hulki*	?	?	?	?	?	0.25	0.49	?	?
*Caiman crocodilus*	?	?	?	?	?	0.3	0.48	?	?
*Cricosaurus araucanensis*	10	5	1	1	0.26	0.18	0.59	1.33	0.8
Crocodylus johnstoni*	6	18	5	8	?	0.28	0.45	0.63	0.45
*Crocodylus niloticus*	?	?	?	?	?	0.29	0.51	?	?
*Dakosaurus cf. andiniensis*	?	?	?	?	?	?	?	?	?
Desmatosuchus spurensis*	?	?	?	?	?	0.29	0.26	?	?
Ebrachosaurus neukami*	5	23	18	8	0.18	0.18	0.53	?	?
Gavialis gangeticus*	9	36	15	22	0.19	0.22	0.38	0.67	0.55
Goniopholis pugnax*	?	?	?	?	?	0.26	0.36	?	?
*Lohuecosuchus megodontos*	6	4	7	5	?	0.18	0.41	1	0.86
*Machaeroprosopus mccauleyi*	?	?	?	?	0.13	0.14	0.51	?	?
*Metriorynchus cf. westermanni*	?	?	?	?	?	?	?	?	?
Parasuchus angustifrons*	6	16	9	11	0.24	0.2	0.49	?	?
*Parringtonia gracilis*	?	?	?	?	?	?	?	?	?
Pelagosaurus typus*	8	9	6	4	0.29	0.26	0.37	0.86	0.6
Pholidosaurus meyeri*	?	?	?	?	?	0.2	0.37	1.33	0.6
*Prestosuchus chiniquensis*	?	?	?	?	?	?	?	?	?
*Rhabdognathus aslerensis*	12	19	4	8	0.29	0.15	0.61	0.5	0.36
*Rukwasuchus yajabalijekundu*	?	?	?	?	?	0.29	0.47	1.14	2
Sebecus icaeorhinus*	?	?	?	?	0.2	0.25	0.38	?	?
Simosuchus clarki*	?	?	?	?	0.43	0.32	0.32	0.56	0.5
*Steneosaurus bollensis*	13	18	5	7	?	0.26	0.34	0.67	0.89
Steneosaurus cf. gracilirostris*	13	38	19	14	?	?	?	1.17	0.82

**Taxon**	**PL:(EL-OL)**	**LW:LH**	**ECL:LH**	**AA:PA**	**AA:LA**	**PA:LA**	**CW:(EL-OL)**
*Aegisuchus witmeri*	?	?	?	?	?	?	?
Alligator mississippiensis*	0.2	0.78	0.44	2.92	2.69	0.92	0.42
*Allodaposuchus hulki*	?	?	?	?	?	?	0.5
*Caimin crocodilus*	?	?	?	?	?	?	0.57
*Cricosaurus araucanensis*	0.26	0.92	0.51	5	5	1	0.45
Crocodylus johnstoni*	0.19	1.08	0.46	3.6	2.25	0.63	0.51
*Crocodylus niloticus*	?	?	?	?	?	?	0.6
*Dakosaurus cf. andiniensis*	?	?	?	?	?	?	?
Desmatosuchus spurensis*	0.14	?	?	?	?	?	0.39
Ebrachosaurus neukami*	0.15	1.29	0.36	1.28	2.88	2.25	0.38
Gavialis gangeticus*	0.12	1	0.43	2.4	1.64	0.68	0.35
Goniopholis pugnax*	?	?	?	?	?	?	0.41
*Lohuecosuchus megodontos*	0.1	0.64	0.55	0.57	0.8	1.4	0.3
*Machaeroprosopus mccauleyi*	?	?	?	?	?	?	0.3
*Metriorynchus cf. westermanni*	?	?	?	?	?	?	?
Parasuchus angustifrons*	0.17	1.29	0.43	1.78	1.45	0.82	0.4
*Parringtonia gracilis*	?	?	?	?	?	?	?
Pelagosaurus typus*	0.28	0.79	0.57	1.5	2.25	1.5	0.42
Pholidosaurus meyeri*	0.23	?	?	?	?	?	0.32
*Prestosuchus chiniquensis*	?	?	?	?	?	?	?
*Rhabdognathus aslerensis*	0.21	1.04	0.48	4.75	2.38	0.5	0.39
*Rukwasuchus yajabalijekundu*	0.07	?	?	?	?	?	0.55
Sebecus icaeorhinus*	0.11	?	?	?	?	?	0.41
Simosuchus clarki*	0.19	?	?	?	?	?	0.46
*Steneosaurus bollensis*	0.09	0.78	0.63	3.6	2.57	0.71	0.4
Steneosaurus cf. gracilirostris*	?	1	0.5	2	2.71	1.36	?

**Taxon**	**Source**
*Aegisuchus witmeri*	Holliday & Gardner (2012)
Alligator mississippiensis*	Witmer & Ridgely (2008)
*Allodaposuchus hulki*	Blanco et al. (2015)
*Caimin crocodilus*	Jirak & Janacek (2017)
*Cricosaurus araucanensis*	Herrera, Leardi & Fernández (2018)
Crocodylus johnstoni*	Witmer et al. (2008)
*Crocodylus niloticus*	Jirak & Janacek (2017)
*Dakosaurus cf. andiniensis*	Herrera & Vennari (2015)
Desmatosuchus spurensis*	Hopson (1979)
Ebrachosaurus neukami*	Lautenschlager & Butler (2016)
Gavialis gangeticus*	Pierce, Williams & Benson (2017)
Goniopholis pugnax*	Edinger (1938)
*Lohuecosuchus megodontos*	Serrano-Martínez et al. (2019)
*Machaeroprosopus mccauleyi*	Holloway, Claeson & O’keefe (2013)
*Metriorynchus cf. westermanni*	Fernández et al. (2011)
Parasuchus angustifrons*	Lautenschlager & Butler (2016)
*Parringtonia gracilis*	Nesbitt et al. (2017)
Pelagosaurus typus*	Pierce, Williams & Benson (2017)
Pholidosaurus meyeri*	Edinger (1938) and Hopson (1979)
*Prestosuchus chiniquensis*	Mastrantonio et al. (2013)
*Rhabdognathus aslerensis*	This study
*Rukwasuchus yajabalijekundu*	Sertich & O’Connor (2014)
Sebecus icaeorhinus*	Colbert, Simpson & Williams (1946) and Hopson (1979)
Simosuchus clarki*	Kley et al. (2010)
*Steneosaurus bollensis*	Herrera, Leardi & Fernández (2018)
Steneosaurus cf. gracilirostris*	Brusatte et al. (2016)

**Notes.**

SW Skull Width at cerebrum CFA Cephalic Flexure Angle PFA Pontine Flexure Angle EL Endocast Length OL Olfactory Length CW Cerebrum Width PW Pituitary Width PH Pituitary Height PL Pituitary Length LH Labyrinth Height LW Labyrinth Width CL Cochlear Length ASCA Anterior Semicircular Canal Area PSCA Posterior Semicircular Canal Area LSCA Lateral Semicircular Canal Area

## Description

The superb three-dimensional preservation of AMNH FARB 33354 and the high resolution nature of the obtained µCT scans resulted in an exquisitely detailed endocast (Fig. 1). The endocranial anatomy of *Rhabdognathus* exhibits a composite of morphological features seen in multiple crocodylomorph taxa as well as several unique features not previously described in crocodylomorphs. Here we divide our description of the cranial endocast into three systems: endocranium, endosseous labyrinth, and paratympanic sinuses.

### Endocranium

**Figure 2:**
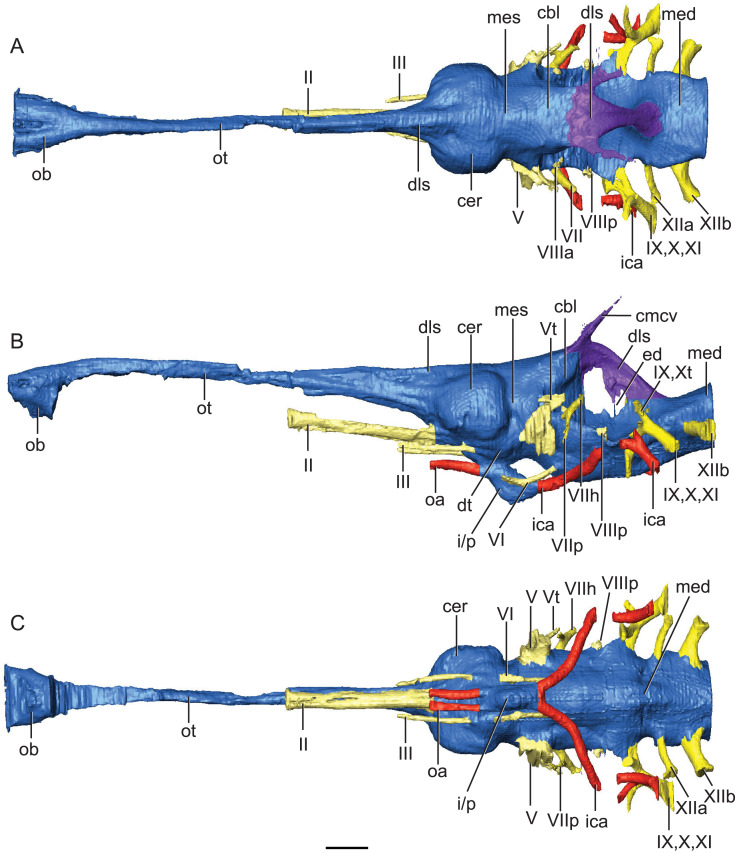
Reconstruction of endocranial neuroanatomy of *Rhabdognathus aslerensis*. (A) Dorsal. (B) Lateral. (C) Ventral. Abbreviations: cbl, cerebellum; cer, cerebrum; cmcv, caudal middle cerebral vein; dls, dorsal longitudinal sinus; dt, dorsal thalamus; ed, endolymphatic duct; ica, internal carotid artery; i/p, infundibulum/pituitary; med, medulla; mes, mesencephalon; oa, ophthalmic artery; ob, olfactory bulb; ot, olfactory tract. Cranial nerves: II, olfactory nerve; III, oculomotor nerve; V, trigeminal nerve; Vt, tympanic branch of trigeminal nerve; VI, abducens nerve; VII, facial nerve; VIIh, hyomandibular branch (chorda tympani) of facial nerve; VIIp, palatal branch of facial nerve; VIIIa, anterior nerve bundle of vestibulocochlear nerve; VIIIp, posterior nerve bundle of vestibulocochlear nerve; IX, glossopharyngeal nerve ; X, vagus nerve; Xt, tympanic fibers of vagus nerve; XI, accessory nerve; XIIa, anterior division of hypoglossal nerve; XIIb, posterior division of hypoglossal nerve. Scale bar equals 10 mm.

The endocranium of *Rhabdognathus* is broadly similar to that of most crocodile-line archosaurs for which there is data (Fig. 2). The brain is relatively straight compared to the anteroventrally directed endocranial space of alligatoroids (Witmer & Ridgely, 2008; Jirak & Janacek, 2017), and phytosaurs (Holloway, Claeson & O’keefe, 2013; Lautenschlager & Butler, 2016). However, it is more S-shaped (more acute cephalic flexion and less acute pontine flexion) than that of thalattosuchians (Brusatte et al., 2016; Pierce, Williams & Benson, 2017; Herrera, Leardi & Fernández, 2018) falling in line with extant taxa such as *Gavialis* (Pierce, Williams & Benson, 2017) and *Crocodylus* (Witmer et al., 2008; Jirak & Janacek, 2017) (Table 2). As noted by Pierce, Williams & Benson (2017), and based on data from birds (Kawabe, Ando & Endo, 2014), the degree of flexion seen in the brain endocast of crocodylomorphs may be due to positioning of the orbits—more laterally positioned orbits like those of thalattosuchians may correlate with less flexed brains endocasts. This suggestion is borne out in *Rhabdognathus* which indeed displays moderate brain endocast flexion and less laterally positioned orbits relative to thalattosuchians.

Starting in the anterior portion of the brain endocast, the olfactory tract is relatively straight and slightly ventrally directed at its most anterior end. This condition exhibits the lack of cephalic flexion seen in thalattosuchians (Brusatte et al., 2016; Pierce, Williams & Benson, 2017; Herrera, Leardi & Fernández, 2018) and most closely resembles the olfactory orientation seen in *Lohuecosuchus* (Serrano-Martínez et al., 2019) and *Gavialis* (Pierce, Williams & Benson, 2017). This is in contrast to *Alligator* (Witmer & Ridgely, 2008), *Parringtonia* (Nesbitt et al., 2017), and *Simosuchus* (Kley et al., 2010), which exhibit greater anteroventral direction of the olfactory tract. Of note here is that while taxa with ventrally directed endocrania have acute degrees of cephalic flexion, the latter condition does not entail the former. The position of the olfactory tract can be such that it is in line with the skull table thus directing the post-olfactory portion of the endocast ventrally. This is the condition seen in the comparative phytosaur taxa (Holloway, Claeson & O’keefe, 2013; Lautenschlager & Butler, 2016), as well as in *Caiman* (Jirak & Janacek, 2017), *Crocodylus* (Witmer et al., 2008; Jirak & Janacek, 2017), *Rukwasuchus* (Sertich & O’Connor, 2014), and *Prestosuchus* (Mastrantonio et al., 2013; Mastrantonio et al., 2019).

**Figure 3:**
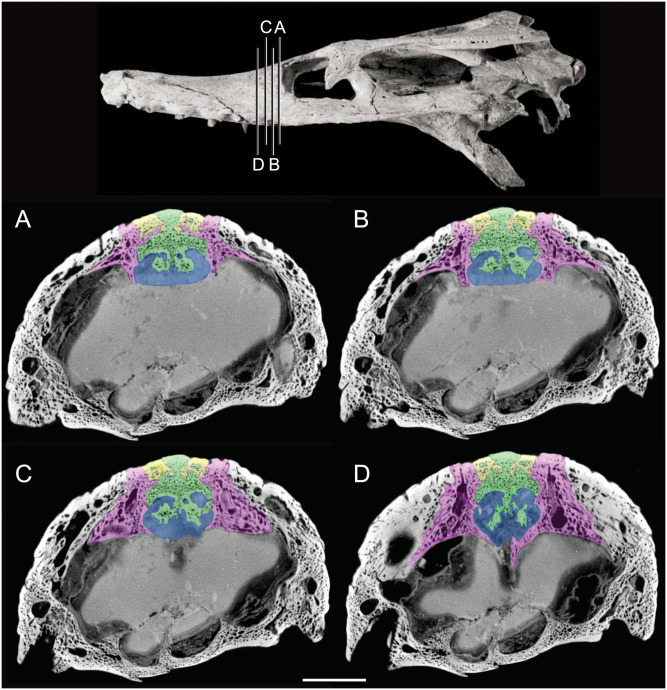
Detail of bony olfactory lamina. Skull of AMNH FARB 33354 at top showing position of coronal CT slices exhibited in A, B, C, and D. Area of olfactory bulb highlighted in blue, frontal in green, nasals in yellow, prefrontals in purple. Scale bar equals 10 mm.

**Figure 4:**
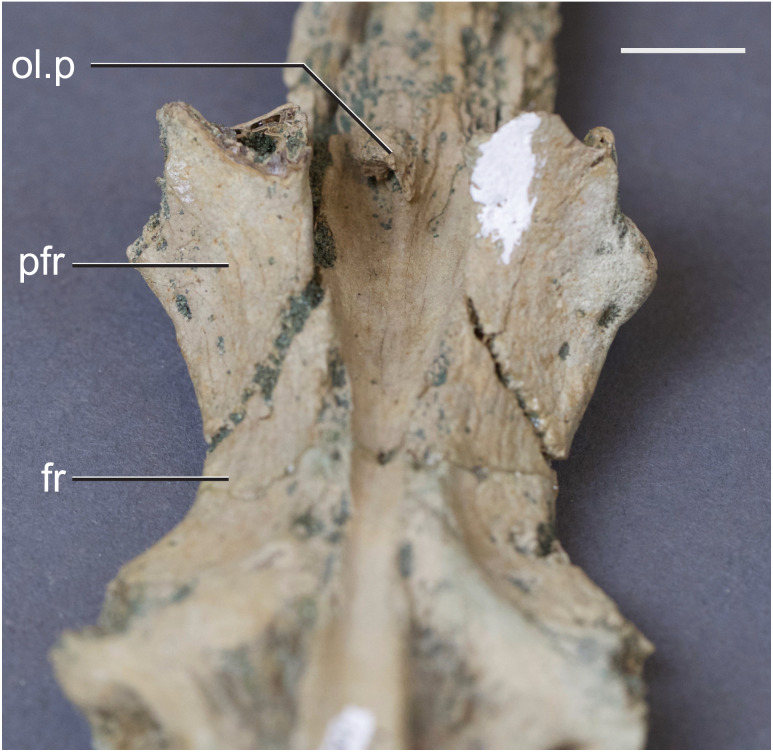
Frontal of *Hyposaurus rogersii* (NJSM 10861). A ventral view showing bony lamina extending from the bone surface. Abbreviations: fr, frontal; ol.p, olfactory process; pfr, prefrontal. Scale bar equals 10 mm.

The olfactory tract in *Rhabdognathus* is undivided as in the thalattosuchian *Cricosaurus araucanensis* (Herrera, Leardi & Fernández, 2018) but unlike *Pelagosaurus* (Pierce, Williams & Benson, 2017). A prominent ridge extends anteriorly along the dorsal surface of the olfactory tract. This ridge is as broad as the remainder of the tract, extends to nearly the midpoint of the tract and is continuous with the dorsal midline surface of the cerebral endocast. This likely represents the anterior most extent of the dorsal longitudinal sinus (Witmer et al., 2008). The olfactory region of *Rhabdognathus* takes the form of a complex expansion bounded dorsally by the frontal bone. In some taxa like *Pelagosaurus*, the olfactory region is divided osteologically by the frontal via a midline crest. In *Simosuchus* (Kley et al., 2010), *Prestosuchus* (Mastrantonio et al., 2013; Mastrantonio et al., 2019), *Rukwasuchus* (Sertich & O’Connor, 2014), the phytosaur *Parasuchus* (Lautenschlager & Butler, 2016), and *Rhabdognathus* there is a partial division of the olfactory region in the endocast. The frontal is pinched between the prefrontals and is unexposed externally in its anteriormost region. It expands ventrally into the cavity of the olfactory region in the form of complex spirals of bone (Fig. 3). Examination of disarticulated cranial remains of *Hyposaurus* (New Jersey State Museum, NJSM 10861) confirms the presence of similar ventral bony projections on the frontal of this taxon too (Fig. 4). *Congosaurus bequaerti* from the Paleocene of Angola (Jouve & Schwarz, 2004) also has a similar morphology suggesting a wider presence among dyrosaurids. We interpret this unique morphology as related to increased surface area for olfaction. The olfactory apparatus is proportionately longer than in other crocodile-line taxa and together comprise about 60% of the total endocast length, a greater proportion than seen in any other taxa with the exception of *Parringtonia* (Nesbitt et al., 2017) in which the post-olfactory endocast appears reduced.

The cerebrum (part of the telencephalon) of *Rhabdognathus* is bulbous and laterally expanded (Fig. 2). However compared to many other taxa, it exhibits a small width relative to the overall length of the endocast (Table 2). The outline of the cerebrum is symmetrical in lateral view, similar to that of the thalattosuchians *Pelagosaurus* (Pierce, Williams & Benson, 2017) and *Steneosaurus* (Wharton, 2000; Brusatte et al., 2016; Herrera, Leardi & Fernández, 2018). These taxa display spherical cerebra, which are dissimilar to extant crocodyloids (Witmer et al., 2008; Witmer & Ridgely, 2008; Dufeau & Witmer, 2015; Jirak & Janacek, 2017), *Simosuchus* (Kley et al., 2010), and *Rukwasuchus* (Sertich & O’Connor, 2014) which tend to exhibit a mediolaterally wide, oval shaped cerebrum. The cerebrum of *Rhabdognathus* is unique among examined crocodylomorphs in that it is nearly square in dorsal view. No midline groove is evident between the cerebral hemispheres, unlike that present in *Pelagosaurus* (Pierce, Williams & Benson, 2017). Instead, *Rhabdognathus* has a prominent dorsal ridge extending from the midpoint of the cerebrum anteriorly along the dorsal surface of the olfactory tract as in *Cricosaurus araucanensis* (Herrera, Leardi & Fernández, 2018). As discussed earlier, we interpret this ridge to be a well-developed anterior portion of the dorsal longitudinal sinus.

**Figure 5:**
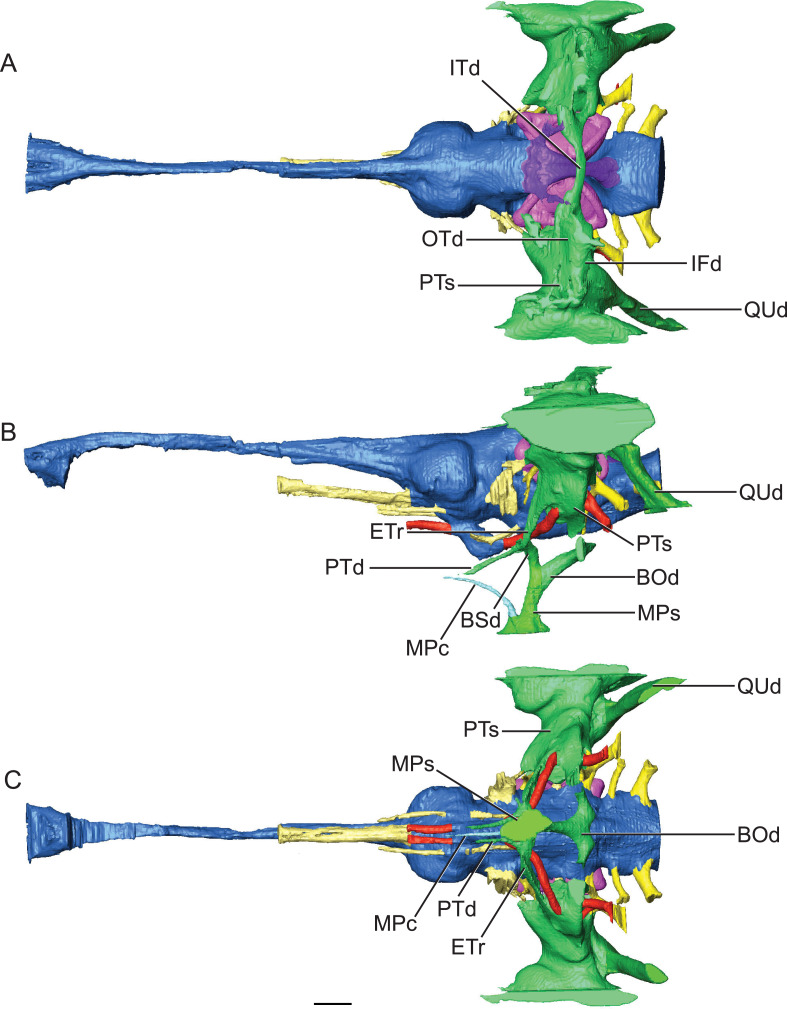
Reconstruction of endocranial morphology and paratympanic system of *Rhabdognathus aslerensis*. (A) Dorsal. (B) Lateral. (C) Ventral. Abbreviations: BOd, basioccipital diverticulum; BSd, basisphenoid diverticulum; ETr, recessus epitubericus; IFd, infundibular diverticulum; ITd, intertympanic diverticulum; OTd, otoccipital diverticulum; MPc, median pterygopharyngeal canal; MPs, median pharyngeal sinus; PTd, pterygoid diverticulum; PTs, pharyngotympanic sinus; QUd, quadrate diverticulum. Scale bar equals 10 mm.

Some structures of the diencephalon appear well visualized in the brain endocast (Fig. 2). Most prominent is the space for the infundibulum and pituitary, which projects posteroventrally from the ventral side of the endocranium inferior to the cerebrum, and a dorsoventrally narrow area that we interpret to be a portion of the dorsal thalamus. This putative dorsal thalamus area is expressed as a slightly raised boss situated between the posteroventral margin of the cerebrum and the anterior edge of the trigeminal ganglion (Fig. 2). The pituitary dimensions are nearest that of extant taxa such as *Gavialis* (Pierce, Williams & Benson, 2017), *Alligator* (Witmer & Ridgely, 2008; Dufeau & Witmer, 2015), and *Crocodylus* (Witmer et al., 2008), as well as the thalattosuchian *Cricosaurus* (Herrera, Leardi & Fernández, 2018), and the notosuchian *Simosuchus* (Kley et al., 2010) (Table 2). Some taxa, primarily thalattosuchians (Brusatte et al., 2016; Pierce, Williams & Benson, 2017; Herrera, Leardi & Fernández, 2018), exhibit an anteroposterior expansion of the pituitary, but this is not the case in *Rhabdognathus*. Associated with the infundibulum/pituitary are paired canals that project anteriorly and posterolaterally from it. The posterior set housed the internal carotid arteries as they entered into the hypophyseal fossa. These canals curve smoothly posterolaterally with a slight dorsal displacement before they are lost into pneumatic space around the inner ear (Fig. 5). In this respect, they are more similar to the internal carotid canal placement in *Pelagosaurus* (Pierce, Williams & Benson, 2017) than to extant crocodylians such as *Gavialis* in which the canals curve dorsolaterally. The anterior set of canals and the orbital arteries they housed (see Brusatte et al., 2016) are positioned more dorsally than where the internal carotid artery enters on the posterior face. This morphology is shared with *Cricosaurus* and *Steneosaurus bollensis* (Herrera, Leardi & Fernández, 2018), whereas other taxa like *Pelagosaurus* (Pierce, Williams & Benson, 2017), and *Steneosaurus* cf. *gracilirostris* (Brusatte et al., 2016) possess a morphology in which the orbital arteries are in line with the internal carotid arteries. In the endocasts of most crocodile-line archosaurs, the orbital arteries are often not present. At the time of their description of *Pelagosaurus* endocranial anatomy, Pierce, Williams & Benson (2017) noted the possibility that presence of orbital artery canals might be a thalattosuchian synapomorphy. Their presence in the dyrosaurid *Rhabdognathus* indicates that this feature might have a broader distribution among crocodylomorphs.

As is typical of crocodile-line archosaurs, the dural envelope of the dorsal longitudinal sinus obscures most of the midbrain (mesencephalon) morphology in the endocast (Fig. 2). The optic lobes are not discernable. However, based on the location of the trigeminal nerve root, we suggest that *Rhabdognathus* has a proportionately anteroposteriorly short midbrain. There is only a narrow exposure of possible midbrain tissue between the trigeminal root, cerebellum, and the otic capsule. This is most similar to juvenile specimens of *Alligator* and adult *Caiman crocodilus* (see Brusatte et al., 2016) and quite dissimilar to the long midbrain region in *Pelagosaurus* (Pierce, Williams & Benson, 2017) and *Cricosaurus* (Herrera, Leardi & Fernández, 2018).

**Figure 6:**
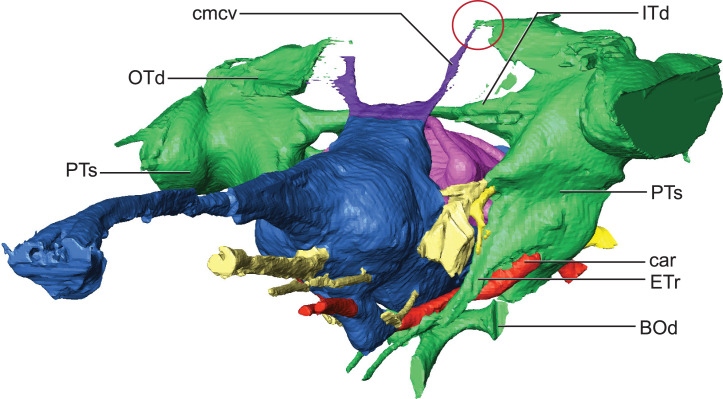
Detail of dorsal longitudinal sinus (DLS) and paratympanic sinuses. Anterolateral close-up view showing communication between DLS, caudal middle cerebral vein, and paratympanic cavity (in the red circle). Abbreviations: BOd, basioccipital diverticulum; car, carotid foramen; cmcv, caudal middle cerebral vein; ETr, recessus epitubericus; ITd, intertympanic diverticulum; OTd, otoccipital diverticulum; PTs, pharyngotympanic sinus.

Hindbrain (rhombencephalon) morphology and the associated cranial nerves and vestibular system are well reflected in the endocast of AMNH FARB 33354 (Fig. 2). The cerebellum is proportionally large as in *Pelagosaurus* and unlike *Gavialis* (see Pierce, Williams & Benson, 2017) and *Caiman crocodilus* (see Brusatte et al., 2016). The cerebellum and the associated dorsal longitudinal venous sinus are dorsally arched and extend well above the level of the cerebrum. This is unlike any crocodylomorph endocast we have examined, although it is closest to thalattosuchian morphology. Two channels project posterodorsally to the left and right from the dorsalmost face of the sinus (Fig. 6). These projections have been interpreted to be for the caudal middle cerebral vein (Pierce, Williams & Benson, 2017; see Witmer & Ridgely, 2008; Porter, 2015; Porter, Sedlmayr & Witmer, 2016) and are present in most thalattosuchians with reconstructed endocasts. In *Rhabdognathus* these projections are especially elongated, forking at their dorsal ends and further expanding into the pneumatic system of the skull, presumably within the parietal. These channels communicate with the paratympanic sinus system (Fig. 6). This communication was previously regarded as a potential synapomorphy of Thalattosuchia by Pierce, Williams & Benson (2017), however, its presence in *Rhabdognathus* in a more exaggerated form suggests that this feature is more broadly distributed in Crocodylomorpha. The otic capsules are greatly enlarged (discussed in more detail below), so much so that they meet along the midline. This provides an unusual demarcation of the posterior boundary of the cerebellum as well as creates a midline venous sinus arch that extends posteroventrally to the brainstem. A projection originating from the ventral surface of this arch lies between the vestibules of the two otic capsules and appears to have functioned as a communication to the subdural space for the drainage of endolymph from the vestibular aqueducts (Fig. 2B). Like the posterior arching of the dorsal longitudinal sinus, capturing this morphology in an endocast seems unique to *Rhabdognathus* as it has not been described in other endocasts.

The remainder of the hindbrain endocast, the medulla, is considerably elongate being over twice the length of the midbrain and cerebrum combined (Table 2). This long medulla is similar to the condition in *Gavialis* (University of Florida, UF 11898) and unlike the relatively shortened medulla of *Pelagosaurus* (Pierce, Williams & Benson, 2017). However, like *Pelagosaurus* it is relatively broad in cross-section, particularly posterior to the vestibular depression where it possesses a distinct lateral bulging prior to the foramen magnum (Fig. 2). There is a ventral swelling on the medulla along the midline up to approximately the midpoint of the vestibular depression.

Due to the exceptional preservation of AMNH FARB 33354, the paths of cranial nerves II, III, V, VI, VII, VIII, IX-XI, and XII could be well segmented. The optic nerves (CN II) appear to share a single extensive bony canal with a partial osteological separation such that two distinct tracts are visible although not entirely separated in two by bone (Fig. 2). The optic nerves are especially elongate compared to nearly all other crocodylomorphs owing to the extreme elongation of the laterosphenoid in dyrosaurids, which is the bone forming the osteological canal for these nerves. The canals from the oculomotor nerves (CN III), which originate from the midbrain ventral and posterior to the optic nerves are not as long as the optic nerve canal and are entirely separated into distinct left and right canals. Compared to the endocasts of other taxa such as *S. bollensis*, the oculomotor nerve pathways are elongate. The length of optic and oculomotor nerve canals represent a potential dyrosaurid synapomorphy resulting from the elongation of the skull, especially the proportionally long laterosphenoid. The abducens nerves (CN VI) are clearly visualized originating from the ventral surface of the anterior portion of the medulla. They travel anteriorly in long canals on either side of the infundibulum/pituitary where upon they exit the skull.

Compared to crown group crocodylians the trigeminal nerve in *Rhabdognathus* is smaller in cross-section and the volume of the trigeminal fossa is lower suggesting a smaller trigeminal ganglion or a ganglion suited farther outside of its osteological borders (George & Holliday, 2013) (Fig. 2). *Pelagosaurus* (Pierce, Williams & Benson, 2017) and the mesoeucrocodylian *Hamadasuchus* (George & Holliday, 2013) also appear to have smaller trigeminal nerves. The divisions of the trigeminal nerve are not apparent in the endocast of AMNH FARB 33354 and the ophthalmic division does not travel in its own canal. What is visualized however is a large branch arising from dorsal aspect of the trigeminal ganglion traveling posterior where it enters into the middle ear cavity. We interpret this to be the “unidentified branch of the trigeminal nerve” (Nx) figured by Hopson (1979) in *Caiman* and subsequently referred to as the tympanic branch (see Witmer et al., 2008). The tympanic branch of the trigeminal is said to travel to the roof of the middle ear cavity where it anastomoses with branches from the glossopharyngeal and vagus nerves (Killian, 1890; Hopson, 1979).

Typically in crocodile-line archosaurs, the facial (CN VII) and vestibulocochlear (CN VIII) nerves arise together as three rami posterior to the trigeminal root and near the anterior margin of the vestibular depression on the medulla (Baird, 1970). The facial nerve emerges most ventrally and the more dorsally situated vestibulocochlear nerve is in fact separate anterior and posterior nerve bundles (CN VIIIa and CNVIIIp). In contrast to this, the facial and vestibulocochlear nerves in *Rhabdognathus* deviate from this largely conserved pattern (Fig. 2B). The facial nerve and CN VIIIa are closely situated but are widely separated from CN VIIIp on the medulla. The root of the facial nerve is located just posterior to the trigeminal nerve. Shortly after emerging from the medulla the facial nerve branches into two, the hyomandibular branch (chordi tympani) that continues in a slight posterodorsal course into the medial ear and a ventrally curving long palatine ramus (Fig. 2B). CN VIIIa leaves the medulla immediately dorsal to the facial nerve root where it can be visualized entering into the large ampulla of the anterior semicircular canal (Fig. 6). CN VIIIp emerges near the midpoint of the vestibular depression half way between the facial nerve and cranial nerves IX, X, and XI. CN VIIIp is short and enters into the base of the cochlear duct (Fig. 2). The glossopharyngeal (CN IX), vagus (CN X), and accessory (CN XI) nerves of *Rhabdognathus* are not distinguishable from each other in the endocast. A single large canal arises from the nerve origins on the medulla and arches posteriorly towards the foramen vagi where it exits. At the apex of the canal a branch comes off that projects anterolaterally carrying the tympanic fibers of the glossopharyngeal and vagus nerves into the middle ear where they anastomose with the tympanic branch of the trigeminal (Killian, 1890).

In *Rhabdognathus*, two large hypoglossal nerves emerge from the posteriormost portion of the medulla. The anterior one (CN XIIa) is roughly half the diameter of the posterior one (CN XIIb). It is not uncommon for the hypoglossal nerve to have multiple roots that often exit through separate foramina (Hopson, 1979; also see Kley et al., 2010). CN XIIa exits the skull through a relatively large foramen immediately medial to the foramen vagi. CN XIIb exits through a slightly larger foramen dorsomedial to the CN XIIa foramen farther out on the neck of the occipital condyle.

### Endosseous labyrinth

**Figure 7:**
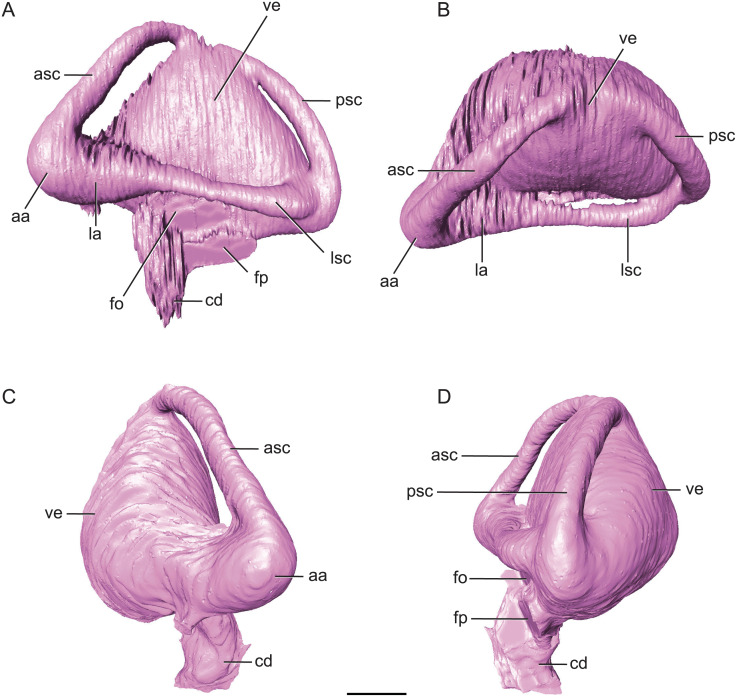
Endossesus labyrinth from left side of *Rhabdognathus aslerensis*. (A) Lateral. (B) Dorsal. (C) Anterior. (D) Posterior. Abbreviations: aa, anterior ampulla; asc, anterior semicircular canal; cd, cochlear duct; fo, foramen ovale; fp, foramen pseudorotundum; la, lateral ampulla; lsc, lateral semicircular canal; psc, posterior semicircular canal; ve, vestibule. Scale bar equals 5 mm.

The inner ear of *Rhabdognathus* exhibits a combination of features shared with extant and extinct genera as well as novel features. The inner ear is incredibly large with a volume nearly the same as the ipsilateral cerebral hemisphere (Fig. 7). The vestibule is significantly expanded essentially obliterating any indication of the common crus of the semicircular canals. The common crus is typically a thin, canal-like structure in other crocodylomorph taxa such as *Steneosaurus*, *Pelagosaurus*, and most extant taxa (Brusatte et al., 2016; Pierce, Williams & Benson, 2017) and leads into the vestibule. In *Rhabdognathus*, the vestibule is extremely bulbous, almost hemispherical. Among extant forms it is closest in morphology to the vestibule in *Gavialis* (see Pierce, Williams & Benson, 2017) which is slightly inflated but not so much that the common crus is not visible. The ampulla of the anterior semicircular canal is the most enlarged of the three ampullae and is continuous with the large ampulla of the lateral semicircular canal. This conformation and relative sizing of the ampullae and canals most closely resembles to the condition in *Gavialis* and *Tomistoma* (Brusatte et al., 2016; Pierce, Williams & Benson, 2017); however, its extent in *Rhabdognathus* is far more exaggerated. The ampullae of the inner ear in *Pelagosaurus* are not particularly large (Pierce, Williams & Benson, 2017).

The ‘M’ shape morphology of the anterior and posterior semicircular canals seen in lateral view in *Gavialis*, *Tomistoma*, *Crocodylus acutus*, *Crocodylus intermedius* (Pierce, Williams & Benson, 2017; Brusatte et al., 2016), and phytosaurs (Lautenschlager & Butler, 2016) in which the anterior and posterior canals are curved along their entire length, is not present in *Rhabdognathus* (Fig. 7). Rather, the semicircular canals display something more similar to the “pyramidal” morphology seen in thalattosuchians (Pierce, Williams & Benson, 2017; Brusatte et al., 2016) and the less curved semicircular canals of *Crocodylus johnstoni* and *Crocodylus moreletii* (Brusatte et al., 2016). In these latter taxa the anterior and posterior canals are relatively straight taking a more direct path to their points of communication with each other and the lateral semicircular canal. This difference in morphology doesn’t seem linked to phylogenetic history and rather displays notable variation at the specific level. The semicircular canals are approximately equal in size with the anterior canal being the longest of the three. Taxa such as *Pelagosaurus* (Pierce, Williams & Benson, 2017), *Steneosaurus* (Brusatte et al., 2016; Herrera, Leardi & Fernández, 2018), and phytosaurs (Lautenschlager & Butler, 2016) exhibit relatively equally proportioned canals, typically with a slightly longer anterior canal. In contrast, extant crocodylians (Brusatte et al., 2016) and possibly *Simosuchus* (Kley et al., 2010) exhibit more substantial asymmetry. Thus it has been put forth that a longer anterior semicircular canal may be a derived feature of Eusuchia or Crocodylia (see Pierce, Williams & Benson, 2017). However, the slightly longer anterior canal in *Rhabdognathus* may be evidence that this feature originated in phylogenetically earlier taxa. Indeed early archosauromorphs such as *Triopticus* and *Trilophosaurus* have noticably longer anterior semicircular canals (Stocker et al., 2016).

The cochlear duct of *Rhabdognathus* is nearly completely delineated in the endocast (Fig. 7). It more closely resembles that of most extant crocodylians in shape but its ventralmost portion may not be present in the endocast (see Brusatte et al., 2016; Pierce, Williams & Benson, 2017). This is in contrast to the elongated ‘paddle-like’ cochlear duct of the thalattosuchians and phytosaurs (Brusatte et al., 2016; Pierce, Williams & Benson, 2017). However, the cochlear duct comprises approximately half the total dorsoventral length of the labyrinth, a proportion closer to that of *Pelagosaurus* (Pierce, Williams & Benson, 2017) and *Steneosaurus* (Brusatte et al., 2016), and slightly greater than extant genera (Pierce, Williams & Benson, 2017; Witmer & Ridgely, 2008; Witmer et al., 2008) and phytosaurs (Lautenschlager & Butler, 2016). The labyrinth overall exhibits a width to height ratio consistent with most crocodile-line archosaurs (∼1.0). This is in contrast to phytosaurs which typically have a proportionally wider inner ear.

The unique aspect of the inner ears of *Rhabdognathus* is obviously their extreme size. They are so large with such inflated vestibules that they contact with each other at the midline posterior to the cerebellum (Figs. 2 and 5). As far as we are aware this has yet to be observed in the endocasts of any other crocodylomorph taxa, but is apparent in the partial braincase of the dyrosaurid *Sokotosaurus* (Natural History Museum United Kingdom, NHMUK R 5616). The dorsal venous sinus arches over the two inner ears, and it projects ventrally to communicate with the medial surfaces of the two vestibules. We interpret this peculiar morphology to be related to the enlargement of vestibules while still maintaining connection to the subdural space for the drainage of endolymph from the vestibular aqueducts (Fig. 2B).

### Paratympanic sinuses

The paratympanic sinus system of *Rhabdognathus* is similar to that of eusuchians such as *Alligator mississippiensis* (Dufeau & Witmer, 2015) and *Aegisuchus witmeri* (Holliday & Gardner, 2012) in overall form. This system in crocodylomorphs can be divided into two systems of separate embryological origin in *Alligator*, the median pharyngeal system and the pharyngotympanic system (Dufeau & Witmer, 2015). A set of older terminology for this system (Owen, 1850; Colbert, Simpson & Williams, 1946) is still frequently in use among crocodylomorph workers but we have adopted the more precise nomenclature advanced by Dufeau & Witmer (2015). Where possible we will refer back to the older names when necessary for clarity with past descriptions.

**Figure 8:**
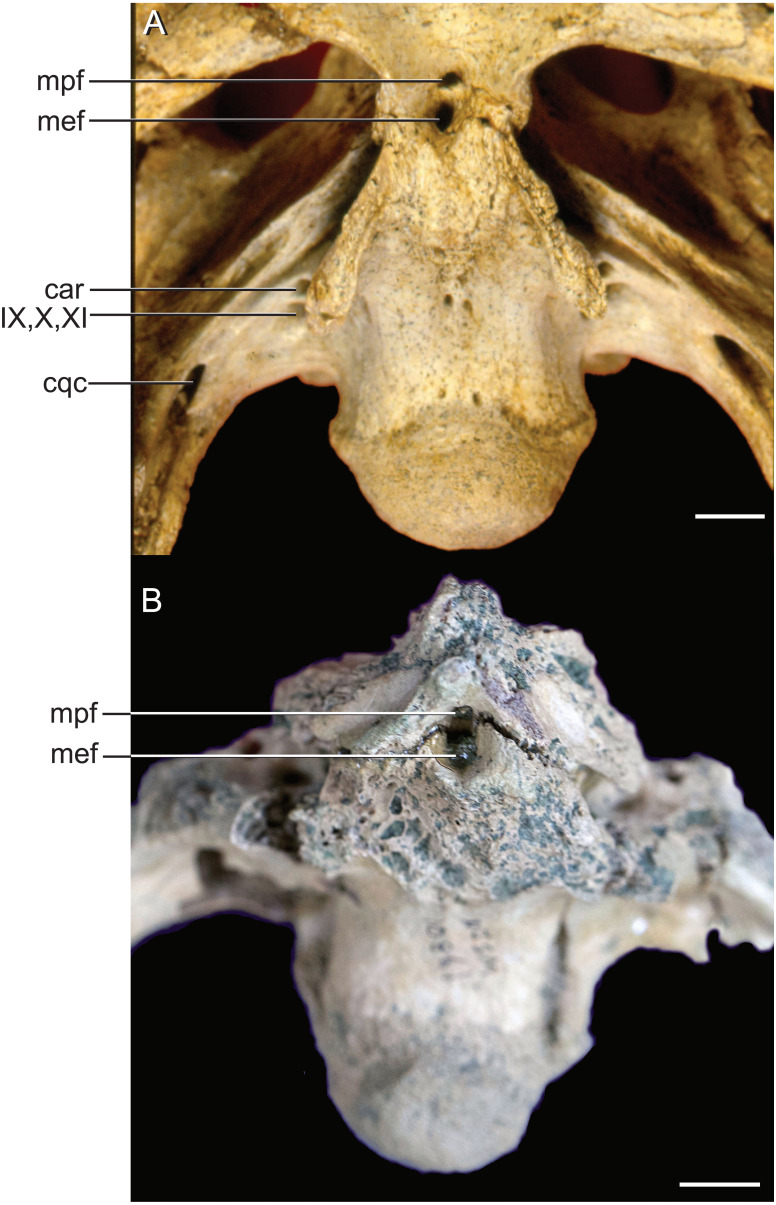
External bony foramina exposed on ventral braincase surface. (A) AMNH FARB 33354 in left ventrolateral view. (B) AMNH FARB 33354 in ventral view. Abbreviations: car, carotid foramen; cqc, cranioquadrate canal foramen; mef, median Eustachian foramen; mpf, median pterygopharyngeal foramen; IX, glossopharyngeal nerve; X, vagus nerve; XI, accessory nerve. Scale bars equal 10 mm.

The median pharyngeal system is composed of the median pharyngeal sinus (= median Eustachian tube; Colbert, Simpson & Williams, 1946) and the basisphenoid diverticulum. The median pharyngeal sinus is a canal that travels roughly dorsoventrally through the suture between the basioccipital and basisphenoid in all crocodyliforms. This canal then branches into the basisphenoid diverticulum anterodorsally (= anterior branch of the median Eustachian tube; Colbert, Simpson & Williams, 1946) and the basioccipital diverticulum posterodorsally (= posterior branch of the median Eustachian tube; Colbert, Simpson & Williams, 1946). The basisphenoid diverticulum bifurcates dorsolaterally. Each diverticula communicates anterodorsally with a recessus epitubaricus (Dufeau & Witmer, 2015) which serves as a link to the pharyngotympanic sinus. When viewed externally, the median Eustachian foramen is divided by a septum in *Rhabdognathus* and *Hyposaurus* (Fig. 8). The CT data for *Rhabdognathus* reveal that the anterior portion of this divided foramen does not communicate with the rest of the median pharyngeal system. Instead, it curves anteriorly through the posterior wall of the pterygoid presumably to communicate with portions of a pterygoid pneumatic cavity within the adductor space (Figs. 5B and 5C). The canal, which can be called the median pterygopharyngeal canal, reflects a previously undocumented feature in a crocodyliform and perhaps is a dyrosaurid apomorphy.

**Figure 9:**
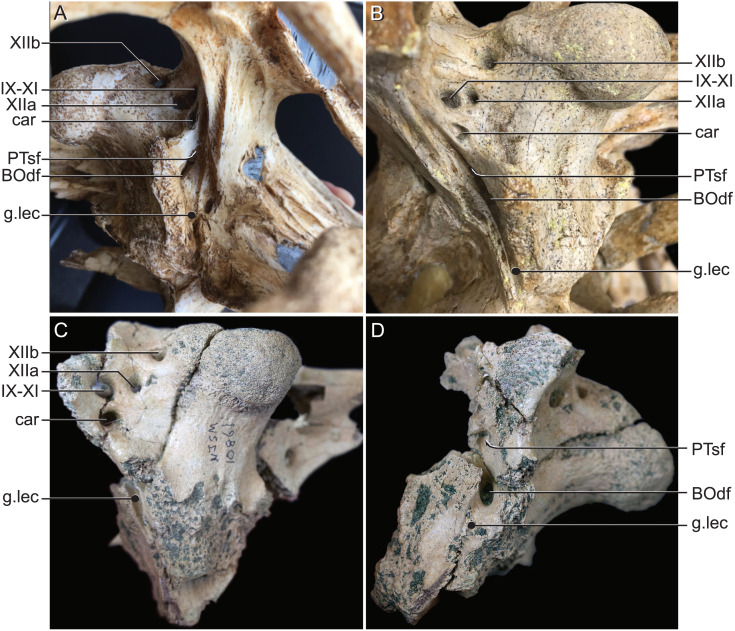
External bony foramina exposed on lateral and posterior braincase walls. (A) AMNH FARB 33354 in left ventrolateral view. (B) AMNH FARB 33354 in right posterolateral view. (C) *Hyposaurus rogersii* NJSM 10861 in left posterolateral view. (D) *Hyposaurus rogersii* NJSM 10861 in left lateral view. Abbreviations: BOdf, foramen for basioccipital diverticulum; car, carotid foramen; g.lec, groove for lateral Eustachian tube; PTsf, pharyngotympanic sinus foramen; IX, glossopharyngeal nerve; X, vagus nerve; XI, accessory nerve; XIIa, anterior bundle of hypoglossal nerve; XIIb, posterior bundle of hypoglossal nerve.

The pharyngotympanic system comprises the rest of the paratympanic sinus with the exception of the tympanum itself. All components of the pharyngotympanic system communicate with the pharynx via the pharyngotympanic sinus. The sinus is paired, with each being lateral to its respective inner ear. Each sinus communicates with a recessus epitubaricus anteroventrally (Fig. 5B). From the recessus epitubaricus project the two pterygoid diverticula anteroventrally. As in *Alligator*, this recess lies between the carotid artery and the trigeminal ganglion. In *Alligator*, a pharyngotympanic tube projects ventrally from the inferior margin of the pharyngotympanic sinus (= rhomboid sinus; Colbert, Simpson & Williams, 1946). This tube is the lateral Eustachian tube. In *Rhabdognathus*, a canal for this tube is absent (Figs. 5B and 5C). Also absent is a direct osteological communication between the pharyngotympanic sinus and the paired basioccipital diverticulum. Rather, the sinus and diverticulum empty into a triangular shaped fossa on the lateral surface of the basioccipital, bounded dorsolaterally by the pterygoid process of the quadrate and posteriorly by the basal tubera of the basioccipital (Fig. 9). Within this fossa a groove is present which appears to connect the sinus and diverticulum outside of the bones of the braincase. This is likely where the pharyngotympanic tube would have been situated and appears to communicate to another fossa externally, as it is only partially delineated by bone. *Pelagosaurus* also lacks bony pharyngotympanic tubes (Pierce, Williams & Benson, 2017) but in the case of this taxon a single clearly demarcated lateral Eustachian foramen is visible on both sides of the braincase (NHMUK 32599). The tubes in *Rhabdognathus* appear to have been proportionally wider anterodorsally and more medially situated than that of *Alligator*. The remainder of the pharyngotympanic sinus is enlarged in *Rhabdognathus*. A short diverticulum invades the dorsal area of the squamosal but does not reach the parietal and fails to communicate with the dorsal projection of the dorsal longitudinal sinus of the brain (unlike *Pelagosaurus* in which a connection is present).

## Discussion

These observations of the *Rhabdognathus* endocranium mark the beginning of a better understanding of dyrosaurid endocranial anatomy and provide important new information about the clade as well as comparative data significant to future work on other crocodylomorphs. Despite the novelty of some of the internal cranial structures, an understanding of the function of these features can be obtained through comparison with other crocodile-line archosaurs. Below we examine some of the unique or highly modified features of *Rhabdognathus*. These include the elongate olfactory tract and expanded olfactory region, the enlargement of the inner ear and resulting communication with the dorsal venous sinus, the external communications of the pharyngotympanic and median pharyngeal sinus, and the novel median pterygopharyngeal canal. Some of these features may reflect neuroanatomical adaptations of dyrosaurids to their predominately near-shore marine habit.

### Paratympanic sinuses

The use of CT visualization has allowed for the improved identification of features previously misinterpreted in this specimen and bears on the interpretation of pharyngotympanic foramina in other closely related taxa. Brochu et al. (2002) interpreted the ventral foramen posterior to the choanae, which is separated into anterior and posterior sections, as the external opening for the anterior and posterior branches of the median Eustachian canal. This makes sense as the opening corresponds topologically to the median Eustachian foramen (median pharyngeal sinus sensu Dufeau & Witmer, 2015). As it turns out, in *Rhabdognathus,* and likely *Hyposaurus*, only the posterior foramen serves as the median Eustachian foramen (Figs. 5 and 8). The anterior portion is a previously unidentified canal that we have named the median pterygopharyngeal canal. The canal arches anteriorly as though to contact the pterygoid diverticula to the left and right of this canal. If these three canals do communicate, this takes place outside of the cranial bones, and thus is not present in the endocast.

The braincase of *Rhabdognathus* has what appears to be a single foramen on the lateral braincase wall, bounded by the basisphenoid anteriorly and the exoccipital posteriorly, that penetrates the basioccipital. Lavocat (1955), Buffetaut (1982), and Brochu et al. (2002) all identified this opening as an unusually located lateral Eustachian foramen. The lateral Eustachian canal typically in crocodyliforms exits the braincase from a ventrally directed foramen. Brochu et al. (2002) noted this condition was distributed more widely among dyrosaurids and in at least some pholidosaurids. Our CT work reveals that this identification of this peculiar morphology is not quite right, but no less bizarre (Fig. 9). What appears to be a single laterally-facing foramen is in fact a deeply recessed fossa that contains two foramina—a ventrally directed foramen in the dorsal portion of the fossa penetrating the otoccipital and a medially directed foramen that indeed penetrates the basioccipital. The dorsal foramen directly communicates with the inferior margin of the pharyngotympanic sinus (= rhomboid sinus; Colbert, Simpson & Williams, 1946), and thus represents the true “lateral Eustachian foramen” although we should note that a true lateral Eustachian canal/tube has not yet formed. This takes place extra-cranially. The laterally-facing foramen is in fact the exit for the basioccipital diverticulum (= the lateral branches of the posterior median canal; Colbert, Simpson & Williams, 1946). Thus we conclude that the basioccipital diverticulum connects with the pharyngotympanic sinus within the fossa on the braincase wall and only at this point does a true lateral Eustachian tube form. Close examination of the basioccipital/basisphenoid contact reveals a hemi-cylindrical depression extending from the lateral fossa that we interpret to be the osteological impression of the lateral Eustachian tubes (Fig. 9). These new osteological interpretations bear on phylogenetic datasets, so we here suggest a new character to capture this novel variation—Lateral Eustachian tube, formation and enclosure in bone: entirely enclosed in bone at its formation from the pharyngotympanic sinus, exiting the skull through a discrete foramen (0); or tube exposed laterally between the basisphenoid/basioccipital suture (1). This would be considered a likely synapomorphy of Dyrosauridae + Pholidosauridae given its current distribution.

### Olfactory apparatus

As is typical of crocodile-line archosaurs, the endocast exhibits an elongated olfactory apparatus. However, in *Rhabdognathus*, the tract and olfactory region comprise two thirds of the total endocast length. This is a morphology which appears to be a further exaggeration of the already generally elongated olfactory tract and bulbs seen in other with longirostrine forms (e.g., phytosaurs and thalattosuchians). This difference in internal structure has an obvious external indication. The skulls of non-dyrosaurid longirostrine taxa, such as *Gavialis* and thalattosuchians, exhibit greater proportional elongation primarily of the nasal cavity bounded by the maxilla and nasals. Despite exhibiting tube-shaped snouts similar to those of dyrosaurids, the braincases of most thalattosuchians and *Gavialis* do not exhibit similar elongation. In dyrosaurids, especially in *Rhabdognathus*, *Hyposaurus*, and *Guarinisuchus*, the most notable elongation is in the post-orbital portion of the skull in the bones enclosing the supratemporal fenestrae (see Brochu et al., 2002). This elongation and its concomitant elongation of the laterosphenoid has a particular effect on the olfactory system and its implications for dyrosaurid ecology are not fully understood. Even in thalattosuchian taxa with particularly enlarged supratemporal openings (e.g., *Cricosaurus araucanensis* (see Herrera, Leardi & Fernández, 2018)) the enlargement is not strictly in the anterior-posterior dimension. Furthermore, there remains a large supratemporal shelf with in the supratemporal fossa formed by the posteriorly inclined postorbital processes of the frontal. The backswept frontal means that the laterosphenoid/frontal contact remains in a more posterior position than it does in derived dyrosaurids (Herrera, Leardi & Fernández, 2018), thus the laterosphenoids in these thalattosuchian taxa are not elongated in the way described here in *Rhabdognathus*. These differences in morphology could imply separate origins for the longirostrine skulls seen in Thalattosuchia and Dyrosauridae. This independent origin would be predicted by the phylogenetic analysis by Wilberg (2015) which places dyrosaurids within Neosuchia and thalattosuchians outside of Crocodyliformes.

McCurry et al. (2017) found that crocodylian skull morphospace correlates best with adaptations for feeding than with phylogenetic ancestry or adaptations for locomotion through a specific habitat. They also found a similar relationship in odontocetes and strong convergence in skull morphology between these lineages. However, the study consisted only of extant taxa and thus the only longirostrine crocodylian examined with similar proportions to a dyrosaurid was the Indian gharial. As previously noted, the crania of dyrosaurids exhibit an elongation of the braincase not seen in extant crocodylians. Despite this, the snout morphology of dyrosaurids likely indicates adaptation for piscivory as piscivorous cetaceans were found to exhibit a similar cranial condition (McCurry et al., 2017). The presence of bony laminae invading the olfactory region has not been described before in any archosaur of which we are aware. We hypothesize this morphology may be related to increased tissue surface area for olfaction analogous to the role some posterior nasal turbinals play in mammalian clades (Adams, 1972). In keeping with the McCurry et al. (2017) study, this modification may be seen as a possible adaptation to near-shore feeding ecology wherein smell may take on a more important component of prey identification.

### Inner ear

In a condition undescribed for any crocodile-line archosaur, the inner ears of *Rhabdognathus* exhibit hyper-expanded vestibules so large that they contact each other at the midline. Additionally, although the semicircular canals show a relatively similar shape pattern as that seen in extant crocodylians, the cross sectional area of the canals are larger. The vestibular system governs the perception of head movements (both linear and rotational acceleration) and mediates eye and neck muscle response to maintain and stabilize posture, balance, and gaze (the vestibulo-ocular and vestibulo-collic reflexes) (Spoor & Zonneveld, 1998). Within the vestibule is the utricle that is one of two otolithic organs which detects linear acceleration and gravitational effects. Furthermore, it is known that increased utricle volume increases sensitivity and performance of the semi-circular canal system (Muller, 1994). We propose that the great enlargement of the vestibule in *Rhabdognathus* would result in a degree of high-speed dynamic locomotor performance in dyrosaurids unlike any other crocodyliform lineage that likely aided an active near-shore predatory ecology. An alternative interpretation of this morphology would be that dyrosaurid taxa such as *Rhabdognathus* were relying on otolith-mediated inner ear function to assist in underwater vibration detection instead of tympanic driven impedance matching.

The cochlear duct lacks some osteological designation ventrally. However, substantial portions of the cochlea were segmented and reveal a morphology more similar to that of extant crocodylians than the proportionally elongated cochlea of thalattosuchians. The cochlear duct of *Rhabdognathus* has a short ventral projection accompanied with a wide lateral face on the lateral sides of each inner ear which hints at the presence of an expansion which is not delineated by bone. This is also present in the endocasts of extant taxa such as *Alligator mississippiensis*, *Tomistoma schlegelii*, *Crocodylus acutus*, and *Crocodylus moreletii* (Brusatte et al., 2016). The proportion of the cochlear length to total endosseous labyrinth height is roughly 0.5 in *Rhabdognathus* which is closest to that of thalattosuchian taxa such as *Pelagosaurus* (= 0.55). Walsh et al. (2009) found that the length of the duct scales roughly with the average best hearing range and frequency of a given taxa. Perhaps, this suggests the hearing capabilities of *Rhabdognathus* may resemble that of thalattosuchian taxa such as *Pelagosaurus* despite differing cochlear morphologies; however, we stress that much work remains to be done on the relationships of cochlear duct morphology to hearing in fossil archosaurs.

### Neuroanatomy and sensory integration

The principle of proper mass states that the size of brain regions reflects processing capacity, thus proportional increases or decreases relate to increases or decreases in function (Jerison, 1973). Thus one would expect larger cerebral regions to be associated with increased interpretation of sensory inputs because of the need for greater neuronal area to execute increasingly complex behaviors (Rogers, 1999). In *Rhabdognathus* we see two sizable increases and modifications to sensory structures, namely the bony lamina which expand surface area in the already enlarged olfactory region, and the extremely enlarged vestibular system of the inner ear. We have interpreted these modifications to be adaptations to prey identification and agile locomotion through aquatic environments. Movement through these environments entail a greater number of sensory inputs to be processed as does integrating sensory inputs for heightened olfaction with the visual system. We see two neuroanatomical features in *Rhabdognathus* that appear to satisfy this prediction. Like thalattosuchians and cetaceans, which exhibit notably larger and more spherical cerebra compared to ancestral lineages, we see a large spherical cerebrum in *Rhabdognathus*. Similarly, we have identified a region on the endocast we think corresponds to the dorsal thalamus. That this region is visualized on the endocast when in other crocodyliforms it is not, suggests an increase in volume in *Rhabdognathus* as well. The dorsal thalamus is generally thought of as a hub or relay point for sensory signals going to the cerebrum.

## Conclusions

Our analysis of the endocranial anatomy of *Rhabdognathus* based on exceptional µCT data yields new morphological and neuroanatomical information that provides important data for understanding the phylogenetic and ecological/behavioral history for dyrosaurids and potentially other closely related crocodyliforms. The expansion of the olfactory region in the form of complex spirals suggests heightened olfaction and its presence may be a potential synapomorphy for Dyrosauridae. The previously confounding laterally-facing lateral Eustachian foramen is now understood to be a complex structure including both a ventrally directed lateral Eustachian foramen and a laterally directed foramen for the basioccipital diverticulum. The formation of a lateral Eustachian tube would have been entirely extra-cranial within the pharynx of *Rhabdognathus* and likely many other dyrosaurid and potentially pholidosaurids as well. Thus we have proposed a new character to capture this morphology. The presence of a delineable exit of the carotid artery on the anterior face of the pituitary fossa in canals which likely housed the orbital artery is a feature present in only some endocasts currently known (namely thalattosuchians) and requires further investigation to understand its phylogenetic spread and possible ecological meaning. The placement of these canals, whether in line or dorsal to those on the posterior face is also of interest. Something found by this study which requires further examination is the distribution of the novel median pterygopharyngeal canal, which may be present in other crocodyliform taxa or may serve as an additional dyrosaurid synapomorphy. Lastly, a suite of neuroanatomical modification suggests adaptation to an agile predatory aquatic ecology in *Rhabdognathus*. These include expanded sense organs (e.g., bony olfactory lamina; hypertrophied vestibule of the inner ear), a possible expansion of the dorsal thalamus, and the clear expansion of the cerebrum to a more symmetrical and spherical shape.

## Additional Information and Declarations

### Data Availability

The following information was supplied regarding data availability: The data is available at MorphoSource:
10.17602/M2/M349173

10.17602/M2/M345754.

## References

[ref-1] Adams DR (1972). Olfactory and non-olfactory epithelia in the nasal cavity of the mouse, *Peromyscus*. American Journal of Anatomy.

[ref-2] Arambourg C (1952). Les vertébrés fossiles des gisements de phosphates (Maroc-Algérie-Tunisie). Notes et Mémoires du Service géologique du Maroc.

[ref-3] Baird IL, Gans C, Parsons TS (1970). The anatomy of the reptilian ear. Biology of the reptilia.

[ref-4] Balanoff AM, Bever GS, Colbert MW, Clarke JA, Field DJ, Gignac PM, Ksepka DT, Ridgely RC, Smith NA, Torres CR, Walsh S, Witmer LM (2016). Best practices for digitally constructing endocranial casts: examples from birds and their dinosaurian relatives. Journal of Anatomy.

[ref-5] Barbosa JA, Kellner AWA, Viana MSS (2008). New dyrosaurid crocodylomorph and evidences for faunal turnover at the K-P transition in Brazil. Proceedings of the Royal Society B: Biological Sciences.

[ref-6] Bergounioux F-M (1956). Les reptiles fossiles des dépôts phosphates sud Tunisiens. Royaume de Tunis Service des Mines, de l’Industrie et de l’Energie. Annales des Mines et de la Geologie.

[ref-7] Blanco A, Fortuny J, Vicente A, Luján ÀH, García-Marçà JA, Sellés AG (2015). A new species of *Allodaposuchus* (Eusuchia, Crocodylia) from the Maastrichtian (Late Cretaceous) of Spain: phylogenetic and paleobiological implications. PeerJ.

[ref-8] Bona P, Carabajal AP, Gasparini Z (2017). Neuroanatomy of *Gryposuchus neogaeus* (Crocodylia, Gavialoidea): a first integral description of the braincase and endocranial morphological variation in extinct and extant gavialoids. Earth and Environmental Science Transactions of the Royal Society of Edinburgh.

[ref-9] Brochu CA, Bouare ML, Sissoko F, Roberts EM, O’Leary MA (2002). A dyrosaurid crocodyliform braincase from Mali. Journal of Paleontology.

[ref-10] Bronzati M, Montefeltro FC, Langer MC (2015). Diversification events and the effects of mass extinctions on Crocodyliformes evolutionary history. Royal Society Open Science.

[ref-11] Brusatte SL, Muir A, Young MT, Walsh S, Steel L, Witmer LM (2016). The braincase and neurosensory anatomy of an Early Jurassic marine crocodylomorph: implications for crocodylian sinus evolution and sensory transitions. The Anatomical Record.

[ref-12] Buffetaut E (1980). Les crocodiliens paléogènes du Tilemsi (Mali): un aperçu systématique. Palaeovertebrata, Mémoire Jubilaire Rene Lavocat.

[ref-13] Buffetaut E (1982). Radiation évolutive, paléoécologie et biogéographie des crocodiliens mesosuchiens. Memoires de la Societé Geologique de France.

[ref-14] Buffetaut E, Lauverjat J (1978). Un crocodilien d’un type particulier dans le Cénomanien de Nazaré (Portugal). Comptes Rendus sommaires des Séances de la Société Géologique de France.

[ref-15] Buffetaut E, Wouters G (1979). *Atlantosuchus coupatezi* n. g. n. sp. un nouveau Dyrosauridé (Crocodylia, Mesosuchia) des phosphates montiens du Maroc. Bulletin Trimestriel de la Société Géologique de Normandie et desAmis du Muséum du Havre.

[ref-16] Colbert EH, Simpson GG, Williams CS (1946). *Sebecus*, representative of a peculiar suborder of fossil Crocodilia from Patagonia. Bulletin of the American Museum of Natural History.

[ref-17] Dufeau DL, Witmer LM (2015). Ontogeny of the middle-ear air-sinus system in *Alligator mississippiensis* (Archosauria: Crocodylia). PLOS ONE.

[ref-18] Edinger T (1938). Über steinkerne von hirn-und ohr-höhlen der mesosuchier *Goniopholis* und *Pholidosaurus* aus dem bückeburger Wealden. Acta Zoologica.

[ref-19] Edinger T (1975). Paleoneurology 1804-1966. An annotated bibliography. Advances in Anatomy, Embryology, and Cell Biology.

[ref-20] Fernández MS, Carabajal AP, Gasparini Z, Chong Díaz G (2011). A metriorhynchid crocodyliform braincase from northern Chile. Journal of Vertebrate Paleontology.

[ref-21] George ID, Holliday CM (2013). Trigeminal nerve morphology in *Alligator mississippiensis* and its significance for crocodyliform facial sensation and evolution. The Anatomical Record.

[ref-22] Godoy PL, Benson R, Bronzati M, Butler R (2019). The multi-peak adaptive landscape of crocodylomorph body size evolution. BMC Evolutionary Biology.

[ref-23] Halstead L (1975). *Sokotosuchus ianwilsoni* n. g. n. sp. a new teleosaur crocodile from the Upper Cretaceous of Nigeria. Journal of Mining and Geology.

[ref-24] Hastings AK, Bloch JI, Cadena EA, Jaramillo CA (2010). A new small short-snouted dyrosaurid (Crocodylomorpha, Mesoeucrocodylia) from the Paleocene of northeastern Colombia. Journal of Vertebrate Paleontology.

[ref-25] Hastings AK, Bloch JI, Jaramillo CA (2011). A new longirostrine dyrosaurid (Crocodylomorpha, Mesoeucrocodylia) from the Paleocene of north-eastern Colombia: biogeographic and behavioural implications for New-World Dyrosauridae. Palaeontology.

[ref-26] Hastings AK, Bloch JI, Jaramillo CA (2015). A new blunt-snouted dyrosaurid, *Anthracosuchus balrogus* gen. et sp. nov. (Crocodylomorpha, Mesoeucrocodylia), from the Palaeocene of Colombia. Historical Biology.

[ref-27] Herrera Y, Leardi JM, Fernández MS (2018). Braincase and endocranial anatomy of two thalattosuchian crocodylomorphs and their relevance in understanding their adaptations to the marine environment. PeerJ.

[ref-28] Herrera Y, Vennari VV (2015). Cranial anatomy and neuroanatomical features of a new specimen of Geosaurini (Crocodylomorpha: Metriorhynchidae) from west-central Argentina. Historical Biology.

[ref-29] Hill RV, MCcartney JA, Roberts E, Bouaré M, Sissoko F, O’Leary MA (2008). Dyrosaurid (Crocodyliformes: Mesoeucrocodylia) fossils from the upper Cretaceous and Paleogene of Mali: implications for phylogeny and survivorship across the K/T boundary. American Museum Novitates.

[ref-30] Holliday CM, Gardner NM (2012). A new eusuchian crocodyliform with novel cranial integument and its significance for the origin and evolution of Crocodylia. PLOS ONE.

[ref-31] Holloway WL, Claeson KM, O’keefe FR (2013). A virtual phytosaur endocast and its implications for sensory system evolution in archosaurs. Journal of Vertebrate Paleontology.

[ref-32] Hopson J (1977). Relative brain size and behavior in archosaurian reptiles. Annual Review of Ecology and Systematics.

[ref-33] Hopson J, Gans C, Parsons T (1979). Paleoneurology. Biology of the reptilia.

[ref-34] Jerison H (1973). Evolution of the brain and intelligence.

[ref-35] Jirak D, Janacek J (2017). Volume of the crocodilian brain and endocast during ontogeny. PLOS ONE.

[ref-36] Jouve S (2007). Taxonomic revision of the dyrosaruid assemblage (Crocodyliformes: Mesoeucrocodylia) from the Paleocene of the Iullemmeden Basin, West Africa. Journal of Paleontology.

[ref-37] Jouve S, Iarochene M, Bouya B, Amaghzaz M (2005). A new dyrosaurid crocodyliform from the Palaeocene of Morocco and a phylogenetic analysis of Dyrosauridae. Acta Palaeontologica Polonica.

[ref-38] Jouve S, Schwarz D (2004). *Congosaurus bequaerti*, a Paleocene dyrosaurid (Crocodyliformes; Mesoeucrocodylia) from Landana (Angola). Bulletin de l’Institut royal des Sciences naturelles de Belgique, Sciences de la Terre.

[ref-39] Kawabe S, Ando T, Endo H (2014). Enigmatic affinity in the brain morphology between plotopterids and penguins, with a comprehensive comparison among water birds. Zoological Journal of the Linnean Society.

[ref-40] Khosla A, Sertich JJ, Prasad GV, Verma O (2009). Dyrosaurid remains from the intertrappean beds of India and the Late Cretaceous distribution of Dyrosauridae. Journal of Vertebrate Paleontology.

[ref-41] Killian G (1890). Die Ohrmuskeln des Krokodiles nebst vorläufigen Bemerkungen über die Homologie des Musculus stapedius und des Stapes. Jenaische Zeitschrift für Naturwissenschaft.

[ref-42] Kley NJ, Sertich JJW, Turner AH, Krause DW, O’Connor PM, Georgi JA (2010). Craniofacial morphology of *Simosuchus clarki* (Crocodyliformes: Notosuchia) from the Late Cretaceous of Madagascar. Journal of Vertebrate Paleontology.

[ref-43] Koken E (1893). Beiträge zur Kenntnis der Gattung *Nothosaurus*. Zeitschrift der Deutschen Geologischen Gesellschaft.

[ref-44] Lautenschlager S, Butler RJ (2016). Neural and endocranial anatomy of Triassic phytosaurian reptiles and convergence with fossil and modern crocodylians. PeerJ.

[ref-45] Lavocat R (1955). Observations anatomiques nouvelles sur le genre de crocodilien *Dyrosaurus* Pomel. Comptes Rendus de l’Academie des Sciences de Paris.

[ref-46] Mastrantonio BM, Belén M, Desojo JB, Schultz C (2019). The skull anatomy and cranial endocast of the pseudosuchid archosaur *Prestosuchus chiniquensis* from the Triassic of Brazil. Acta Palaeontologica Polonica.

[ref-47] Mastrantonio BM, Schultz CL, Desojo JB, Garcia JB (2013). The braincase of *Prestosuchus chiniquensis* (Archosauria: Suchia). Geological Society, London, Special Publications.

[ref-48] McCurry MR, Evans AR, Fitzgerald EMG, Adams JW, Clausen PD, McHenry CR (2017). The remarkable convergence of skull shape in crocodilians and toothed whales. Proceedings of the Royal Society B: Biological Sciences.

[ref-49] Melstrom K, Irmis RB (2019). Repeated evolution of herbivorous crocodyliforms during the Age of Dinosaurs. Current Biology.

[ref-50] Muller M (1994). Semicircular duct dimensions and sensitivity of the vertebrate vestibular system. Journal of Theoretical Biology.

[ref-51] Nesbitt SJ, Stocker MR, Parker WG, Wood TA, Sidor CA, Angielczyk KD (2017). The braincase and endocast of *Parringtonia gracilis*, a Middle Triassic suchian (Archosaur: Pseudosuchia). Journal of Vertebrate Paleontology.

[ref-52] Owen R (1850). XXVII. On the communications between the cavity of the tympanum and the palate in the Crocodilia (gavials, alligators and crocodiles). Philosophical Transactions of the Royal Society of London.

[ref-53] Pierce SE, Williams M, Benson RBJ (2017). Virtual reconstruction of the endocranial anatomy of the early Jurassic marine crocodylomorph *Pelagosaurus typus* (Thalattosuchia). PeerJ.

[ref-54] Pomel A (1894). Découverte de champsosauriens dans les gisements de phosphorite du suessonien de l’Algérie. Comptes Rendu de l’Académie des Sciences.

[ref-55] Porter WR (2015). Physiological implications of dinosaur cephalic vascular systems. D. Phil. Thesis.

[ref-56] Porter WR, Sedlmayr JC, Witmer LM (2016). Vascular patterns in the heads of crocodilians: blood vessels and sites of thermal exchange. Journal of Anatomy.

[ref-57] Rogers SW (1999). *Allosaurus*, crocodiles, and birds: evolutionary clues from spiral computed tomography of an endocast. The Anatomical Record.

[ref-58] Salih KAO, Evans DC, Bussert R, Klein N, Nafi M, Müller J (2016). First record of *Hyposaurus* (Dyrosauridae, Crocodyliformes) from the Upper Cretaceous Shendi Formation of Sudan. Journal of Vertebrate Paleontology.

[ref-59] Serrano-Martínez A, Knoll F, Narváez I, Lautenschlager S, Ortega F (2019). Inner skull cavities of the basal eusuchian *Lohuecosuchus megadontos* (Upper Cretaceous, Spain) and neurosensorial implications. Cretaceous Research.

[ref-60] Sertich JJW, O’Connor PM (2014). A new crocodyliform from the middle Cretaceous Galula Formation southwestern Tanzania. Journal of Vertebrate Paleontology.

[ref-61] Shiller TA, Porras-Muzquiz HG, Lehman TM (2016). *Sabinosuchuscoahuilensis*, a new dyrosaurid crocodyliform from the Escondido Formation (Maastrichtian) of Coahuila, Mexico. Journal of Vertebrate Paleontology.

[ref-62] Spoor CF, Zonneveld FW (1998). Morphometry of the primate bony labyrinth: a new method based on high-resolution computed tomography. Journal of Anatomy.

[ref-63] Stocker MR, Nesbitt SJ, Criswell KE, Parker WG, Witmer LM, Rowe TB, Ridgely R, Brown MA (2016). A dome-headed stem archosaur exemplifies convergence among dinosaurs and their distant relatives. Current Biology.

[ref-64] Swinton WE (1930). On fossil reptilia from Sokoto Province. Geological Survey of Nigeria Bulletin.

[ref-65] Troxell EL (1925). *Hyposaurus*, a marine crocodilian. American Journal of Science.

[ref-66] Walsh SA, Barrett PM, Milner AC, Manley G, Witmer LM (2009). Inner ear anatomy is a proxy for deducing auditory capability and behaviour in reptiles and birds. Proceedings of the Royal Society B: Biological Sciences.

[ref-67] Wenz S (1968). Contribution à l’étude du genre *Metriorhynchus*. Crâne et moulage endocrânien de *Metriorhynchus superciliosus*. Annales de Paléontologie (Vertébrés).

[ref-68] Wharton DS (2000). An enlarged endocranial venous system in *Steneosaurus pictaviensis* (Crocodylia: Thalattosuchia) from the Upper Jurassic of Les Lourdines, France. Comptes Rendus de l’Académie des Sciences - Series IIA - Earth and Planetary Science.

[ref-69] Wilberg EW (2015). What’s in an Outgroup? The impact of outgroup choice on the phylogenetic position of Thalattosuchia (Crocodylomorpha) and the origin of Crocodyliformes. Systematic Biology.

[ref-70] Wilberg EW, Turner AH, Brochu CA (2019). Evolutionary structure and timing of major habitat shifts in Crocodylomorpha. Scientific Reports.

[ref-71] Witmer LM, Ridgely RC (2008). The paranasal air sinuses of predatory and armored dinosaurs (Archosauria: Theropoda and Ankylosauria) and their contribution to cephalic structure. The Anatomical Record.

[ref-72] Witmer LM, Ridgely RC, Dufeau DL, Semones MC, Endo H, Frey R (2008). Using CT to peer into the past: 3D visualization of the brain and ear regions of birds, crocodiles, and nonavian dinosaurs. Anatomical imaging: towards a new morphology.

